# Education Research: Current State and Effectiveness of EEG Education for Residents: A Systematic Review and Meta-Analysis

**DOI:** 10.1212/NE9.0000000000200214

**Published:** 2025-04-09

**Authors:** Jianhua Chen, Si Chen, Yi-Cheng Zhu

**Affiliations:** 1 Department of Neurology, Peking Union Medical College Hospital, Chinese Academy of Medical Sciences and Peking Union Medical College, Beijing, China;; 2 Department of Anesthesiology, Peking Union Medical College Hospital, Chinese Academy of Medical Sciences and Peking Union Medical College, Beijing, China; and; 3 Department of Neurology, State Key Laboratory of Complex Severe and Rare Diseases, Peking Union Medical College Hospital, Chinese Academy of Medical Sciences and Peking Union Medical College, Beijing, China.

## Abstract

**Background and Objectives:**

EEG interpretation is crucial for residents. However, studies indicated poor competency among residents in interpreting EEGs. We conducted this systematic review and meta-analysis to assess the current state and effectiveness of EEG education for residents.

**Methods:**

A literature search was conducted in electronic bibliographic databases including PubMed, EMBASE, Web of Science, and Cochrane Library. Official websites of organizations including the International League Against Epilepsy, American Clinical Neurophysiology Society, American Epilepsy Society, International Child Neurology Association, Canadian Society of Clinical Neurophysiologists, and China Association Against Epilepsy were also searched for information on EEG education. The Preferred Reporting Items for Systematic Reviews and Meta-analyses methodologic standards for reporting systematic reviews were followed. The inclusion criteria of the articles were those that evaluated the effectiveness of EEG education or surveyed the status of EEG training for residents. Methodologic quality was assessed using the Risk of Bias in Nonrandomized Studies of Interventions, Version 2, tool. A meta-analysis of eligible studies was performed to compare precurriculum and postcurriculum test scores.

**Results:**

A total of 28,024 articles were identified of which 38 were included in the systematic review and 14 in the meta-analysis. EEG education methodologies varied widely. EEG curricula covered a range of topics, from fundamentals and advancements in EEG technology and interpretations to signal analysis, seizure semiology, and clinical applications for patients with epilepsy. There was a significant improvement in posttest scores compared with pretest scores, with a pooled standardized mean difference (SMD) of 1.76 (95% CI 1.14–2.39, *p* < 0.00001). A subgroup analysis of neurology residents, based on 8 studies, showed a pooled SMD of 0.97 (95% CI 0.73–1.22, *p* < 0.00001).

**Discussion:**

EEG education significantly improves residents' test scores and enhances their diagnostic skills of seizures. Future research should focus on evaluating the long-term retention of EEG knowledge following training, particularly regarding specialties.

## Introduction

EEG interpretation is crucial not only for neurology residents but also for those in pediatrics, neonatology, intensive care, neurosurgery, psychology, anesthesiology, and emergency medicine. Despite the well-formulated EEG education milestones for neurology residents proposed by the Accreditation Council for Graduate Medical Education (ACGME), current studies indicate poor competency among residents in accurately interpreting EEGs.^1-3^ Most EEGs are interpreted by neurologists without clinical neurophysiology or epilepsy fellowship training.^4-6^ In fact, over half of graduating adult neurology residents reported a lack of confident in reading EEGs independently.^2,7,8^ This issue is even more prominent among residents in other specialties. Among practicing pediatric neurologists, 76% treat children with seizures or epilepsy and 65% interpret EEGs, yet more than half received their EEG training solely during residency.^3^ Misinterpretation of EEGs can lead to incorrect diagnoses and unnecessary treatments.^2^ Therefore, providing robust EEG education during residency is critically important. To assess the current state and effectiveness of EEG training for residents, we conducted this systematic review and meta-analysis.

## Methods

### Databases and Search Strategy

We conducted the literature search using the following terms: “EEG,” “electroencephalography,” “electroencephalogram,” “electroencephalograms” and “education,” “medical,” “training,” “teaching,” “curriculum,” “curricula,” “course,” “learning,” “workshop,” “school,” “class.” Electronic bibliographic databases including PubMed, EMBASE, Web of Science, and Cochrane Library were systematically and independently searched from January 2019 to January 2024. Potentially eligible studies and the reference lists of the selected articles were examined through manual search. The official websites of the International League Against Epilepsy (ILAE), the American Clinical Neurophysiology Society (ACNS), American Epilepsy Society (AES), the International Child Neurology Association (ICNA), the Canadian Society of Clinical Neurophysiologists (CSCN), and the China Association Against Epilepsy (CAAE) were also searched for EEG education information. The methodologic standards outlined in the Preferred Reporting Items for Systematic Reviews and Meta-analyses guidelines for reporting systematic reviews, and the stepwise approach for conducting systematic reviews in medical education were followed.^9,10^ The present protocol was registered online at the International Prospective Register of Systematic Reviews (crd.york.ac.uk/prospero, registration number: CRD42024558277).

### Study Selection

The inclusion criteria of the studies were those that evaluated the effectiveness of EEG education or surveyed the status of EEG education for residents. Exclusion criteria included studies that did not focus on EEG education for residents, but rather on EEG education for medical students, EEG technologists, nurses, fellows, attending physicians, or those with higher titles. Two independent investigators reviewed the titles and abstracts, retrieving those that met the inclusion criteria for full-text assessment. Articles not published in English were excluded. Studies for meta-analysis were excluded if they lacked detailed data or primarily focused on nonresidents. Any discrepancies in these procedures were resolved through discussion among all authors until a consensus was reached.

### Data Extraction

Data were extracted by 2 independent reviewers and verified by a third reviewer. The following information was extracted: first author and publication year, content and duration of the EEG curriculum, EEG trainees, number of EEGs interpreted, and the mean and standard deviation of percent correct scores for precurriculum and postcurriculum tests.

### Study Quality and Risk of Bias Assessment

Three reviewers independently evaluated the studies' quality. The quality of the included studies was assessed using the NIH Quality Assessment Tool for Before-After Studies, with ratings categorized as “good,” “fair,” or “poor.”^11^ Methodologic quality was assessed using the Risk Of Bias in Nonrandomized Studies of Interventions, Version 2, tool, with results visualized according to the corresponding risk-of-bias assessment methodology.^12^

### Statistical Analyses

A meta-analysis of all eligible studies was conducted by a random-effects model using Review Manager 5.3 software (The Cochrane Collaboration). Precurriculum and postcurriculum test scores were analyzed as continuous variables. The pooled standardized mean differences (SMDs) with 95% CIs between precurriculum and postcurriculum test scores were calculated. Statistical heterogeneity was assessed using the *I*^2^ and τ^2^ statistics and visual inspection of the forest plots. An *I*^2^ ≥75% or a *p* value <0.05 was interpreted as considerable heterogeneity. Sensitivity analysis was conducted by removing each study individually to assess the quality and consistency of the results.

### Data Availability

Data will be provided to other investigators on request made to the corresponding author, YC Zhu, in accordance with International Committee of Medical Journal Editors requirements.

## Results

**Figure 1:**
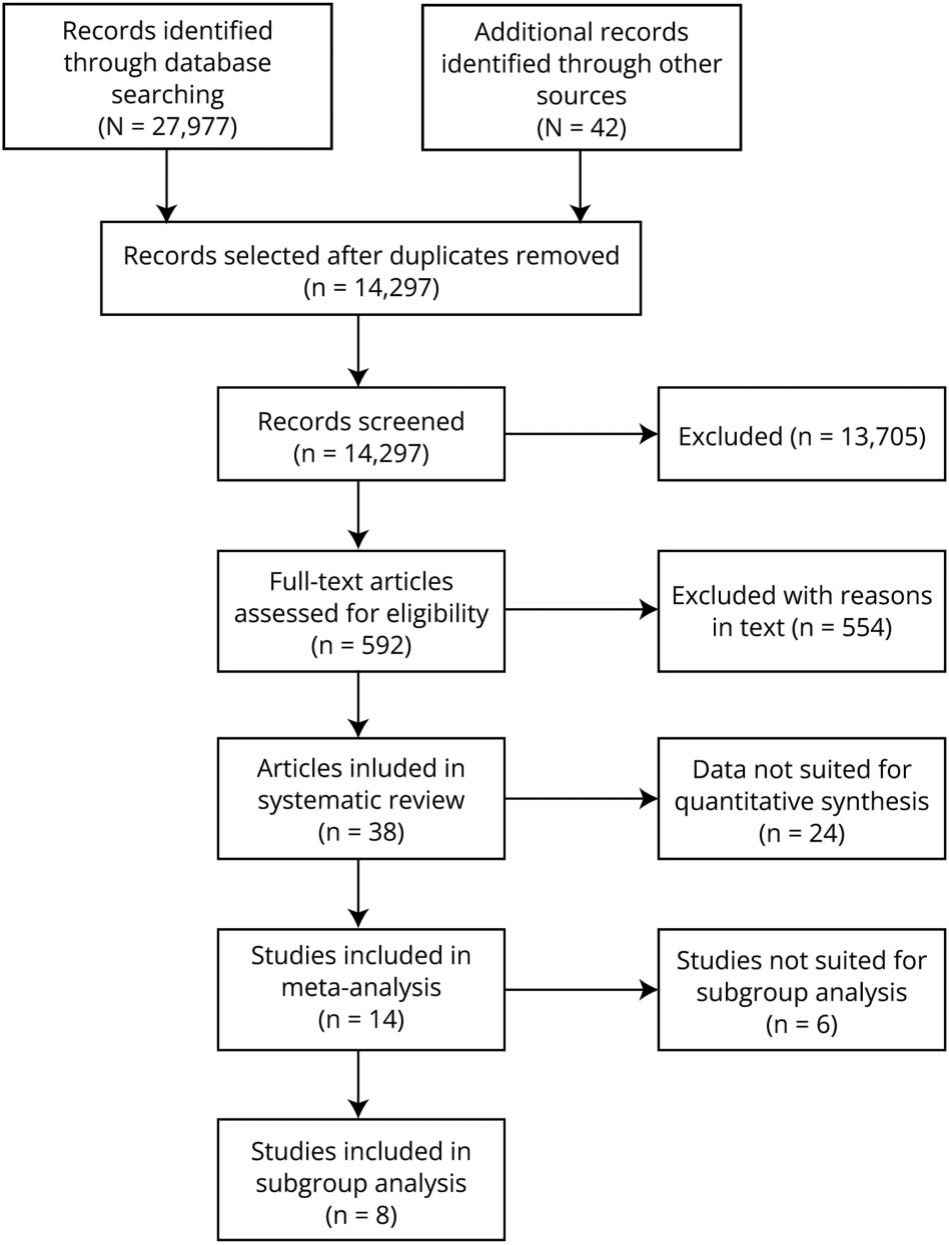
Flowchart of the Selection of Studies

A total of 28,024 articles were screened, and 38 met the inclusion criteria for the systematic review.^1-3,5-8,11,13-42^ Of these, 14 studies were included in the meta-analysis which evaluated the effectiveness of EEG education for residents.^14-19,21,23-29^ Figure 1 outlined the search and selection process. The characteristics of included studies are summarized in Table 1.

**Table 1:** Studies on EEG Education for Residents With Assessments

Study	Teaching method	EEG trainees	Duration of curriculum	Contents of curriculum	No. of EEGs interpreted	Precurriculum tests scores	Postcurriculum tests scores
Fahy et al., 2019^14^	An interdisciplinary module within the anesthesiology residents program	Anesthesiology residents	A 1-mo rotation with weekly 1-h EEG sessions	Basics of monitoring, EEG physiology, and clinical applications	At least 14–15 EEGs, averaging 24	35.6% ± 14.77%	83.3% ± 10.42%
Jenkinson et al., 2020^16^	A didactic lecture, a written workbook, and 2 tutorials of the written workbook	Trainees in a neonatal ICU	NR	A structured aEEG education programme	NR	49.58% ± 7.78%	82.83% ± 2.63%
Yadala et al., 2020^17^	Live, interactive EEG sessions on Zoom	Neurology residents	Eight weeks	NR	NR	52.17% ± 23.81%	79.84% ± 11.87%
Fahy et al., 2021^19^	An online, interactive EEG interpretation simulator	Neurology residents	NR	Normal EEG, artifact; focal seizures, burst suppression, medication effect, focal epileptogenic potentials, slowing, coma, postanoxic myoclonus, cerebral ischemia, sleep/sedative effects, primary generalized epilepsy, brain death, NCSE	Ten scenarios with scrolling EEGs	61.2% ± 7.7%	66.1% ± 7.9%
Pan et al., 2021^18^	EEG interpretation at noon conferences	Neurology residents	Three noon conferences	ACNS standardized critical care EEG terminology, unified EEG criteria for NCSE	48 EEGs	46.71% ± 24.86%	67.20% ± 7.60%
Legriel et al., 2021^21^	A face-to-face EEG course supplemented by e-learning	ICU residents, senior physicians, fellows, medical students, and nurses	90 d with four 90-min face-to-face courses and e-learning sessions	Theoretical notions, illustrative EEG traces, and training tests followed by illustrated and commented answers	Ten 23-s EEG epochs	30% ± 14.81%	80% ± 14.81%
Berger et al., 2022^23^	An electronic curriculum with 4 interactive module	Anesthesiology residents	6-mo study period	EEG spectrogram interpretation and its use in anesthetic titration	159 cases	60% ± 21.8%	90% ± 21.8%
Asukile et al., 2022^25^	An interactive, multimodal web-based EEG program	Neurology residents, neurologists, other medical doctors, and medical technologists	Six months	Neurophysiologic basis, signal processing, electrical safety, normal/abnormal waveforms, artifacts, and clinical use in epilepsy and encephalopathy management	125 EEGs	46.7% ± 17.6%	64.1% ± 18%
Kural et al., 2022^24^	Two training sessions and a teaching module	Neurology residents, neurophysiology trainees	Two sessions, 3 mo apart	EEGs in 2 successive sessions, with the second preceded by a teaching module on IFCN criteria	70 EEGs	64.29% (58.57%–68.57%)	81.43% (77.14%–87.14%)
Nascimento et al., 2022^26^	Didactic EEG lectures	Adult neurology residents	Four 60-min lectures with a 1-mo EEG rotation	Introduction to EEG, artifacts, normal/abnormal EEG, ICU EEG, and ictal/interictal EEG findings	Over 40 EEGs in one-third of programs, 0–10 in 14% of programs	47.35% ± 20.86%	59.65% ± 23.20%
Nascimento et al., 2022^27^	Prerecorded video didactics and candidate IEDs with instant feedback	Junior adult and pediatric neurology residents	30 d	Six IFCN criteria for IED identification	500 10s-EEGs	69.69% ± 5.11%	76.54% ± 3.84%
Yuan et al., 2022^28^	EEG lectures, self-learning videos, online modules, and intraoperative teaching	Pediatric anesthesia residents, new staff, clinical fellows, and nurse anesthetists	Over 12 mo	EEG-guided anesthesia, propofol pharmacology, EEG waveforms, spectral edge frequency, and density spectral array	At least 10 EEG-TIVA cases	37.8% ± 23.2%	59.3% ± 17.6%
Passiak et al., 2023^3^	EEG lectures, epilepsy-related conferences, self-directed modules, and supplemental educational material	Adult and pediatric neurology residents	4-wk	Electrode placement, electrophysiology and polarity, artifacts, EEG background; normal/abnormal variants, epileptiform abnormalities, ictal/interictal EEG, neonatal EEG; advanced clinical vignettes, rhythmic and periodic epileptiform abnormalities	118–147 slides	60.0% ± 12.9%	77.9% ± 11.8%
Mohammadi et al., 2023^29^	In-person and virtual education	Pediatric neurology residents	Two 1-h sessions held biweekly	Benign variants	NR	46.49% ± 16.48%	77.54% ± 14.62%

Abbreviations: ACNS = American Clinical Neurophysiology; aEEG = amplitude integrated EEG; ICU = intensive care unit; IED = interictal epileptiform discharge; IFCN = International Federation of Clinical Neurophysiology; NCSE = nonconvulsive status epilepticus; NR = not reported; TIVA = total intravenous anesthesia.

### Methodology of EEG Education

Residents usually receive EEG education through EEG training and their rotations in epilepsy, clinical neurophysiology, or critical care.^5,18,32^ EEG training uses diverse methods, including didactic lectures, self-guided study, interactive web-based training simulator, and video-based resources. Although face-to-face lectures have been the traditional teaching model for EEG training, virtual EEG education and online lectures became especially popular during the coronavirus disease 2019 pandemic.^17,18^ Most EEG training programs use a combination of 2 or more of these teaching methods.^22^

Standardized terminology can be used in EEG training for residents. The ACNS recommends using the ACNS Critical Care EEG terminology to describe EEG patterns in critically ill patients.^15^ Adult neurology residents can be effectively trained in this terminology and become proficient enough to pass the certification examination offered by the ACNS.^15^ Implementing the International Federation of Clinical Neurophysiology (IFCN) criteria for identifying interictal epileptiform discharges (IEDs) can improve residents' diagnostic performance and interrater agreement.^24^

### EEG Curriculum Content

The EEG curriculum covers a wide range of topics, including the fundamentals and advancements in EEG technology, interpretations techniques, signal analysis, seizure semiology, and clinical applications for managing patients with epilepsy. These curricula are tailored to the needs of various specialties and differ significantly in focus, methods, and outcomes. Some curricula emphasize theoretical notions and basic EEG knowledge. Others focus on specific topics such as EEG terminology, criteria for identifying IEDs, or nonconvulsive status epilepticus.^15,20,22,27^ Residents and EEG technologists usually have very limited face-to-face interaction.^32^ Only a minority of programs include scalp electrode placement and EEG recording process training.^3,32-34^ For anesthesiology residents, the curriculum emphasizes the application of EEG in intraoperative settings, focusing on interpreting EEG patterns affected by anesthetic drugs and understanding the effects of anesthesia on epileptiform activities, particularly EEG spectrogram interpretation to aid in anesthetic titration.^14,23,28^ For ICU residents, the curriculum includes normal findings, common artifacts, the effects of sedation, burst-suppression patterns, paroxysmal EEG patterns, periodic discharges, rhythmic activities, ACNS terminology, and quantitative EEG.^20^ Neurology residents' curricula focus on identifying and interpreting EEG patterns to diagnose neurologic conditions, especially seizures and status epilepticus. A well-outlined list of “must-know” EEG findings with weighted mean scores based on EEG/epilepsy expert scoring is available for both adult and child neurology residents.^27^ The ILAE Education Task Force's “Roadmap to EEGs” offers detailed modules with topics, content, and learning objectives for residents.^1,31^

### EEG Curricula Participants

EEG curricula trainees include residents from Adult and Pediatric Neurology, Anesthesiology, Psychology, Pediatrics, General Medicine, Neurosurgery, Emergency Medicine, and Intensive Care Units. Trainers are usually certified EEG technologists, epilepsy/neurology attending physicians, or fellows.^5,15,17,19,20,30^ Most participants are postgraduate year (PGY) 1–4 residents.^16-18,22,25-27^ In the United States, EEG rotations are typically completed by PGY-2 residents in 50% of programs and by PGY-3 residents in 41% of programs.^30^ PGY-1 and PGY-4 residents typically rotate through EEG in 2% and 7% of programs, respectively.^30^ Some programs also offer additional rotations for PGY-5 residents.^3,32^ In China, EEG rotations are generally assigned to neurology residents in PGY-2 to PGY-4. In Canada, adult neurology programs last 5 years, with residents ranging from PGY-1 to PGY-5.^15^ This differs slightly from the United States, where pediatric neurology residents complete 5 years of training, while adult neurology residents complete 4 years of training. Some participants in the online educational resources are at the PGY-6 level or above.^31,40^

### Duration of EEG Programs

The duration of EEG training for residents varied greatly, ranging from several hours to 6 months. In the United States in 2007, the average duration of EEG rotations for a typical neurology resident was 1.5 months (range: 0–4 months).^43^ By 2021, this had increased to 1.7 months (range: 0–4 months), with zero to 30 EEGs per rotation.^30^ The number of EEGs read during a typical EEG rotation also varied widely, from more than 40 EEGs in about one-third of programs to 0–10 EEGs in about 14% of programs.^30^ In all Canadian residency programs, a 2-month rotation in EEG/epilepsy is a core and mandatory component of residency training.^15^ At McGill University, EEG training for adult neurology residents is integrated with clinical epilepsy training.^44^ In China, the average duration of EEG rotation for adult neurology residents is typically 1 month, sometimes 2 months, which is combined with electromyography and evoked potential rotation.^45^ It was recommended that neurology residents to undergo compulsory extended EEG education of more than 8 weeks of EEG training before graduation.^35^ The more EEG training sessions residents participated in, the more positive clinical behavioral changes were observed, regardless of their postgraduate year or the size of the hospital.^22^

### Learning Outcomes

The first International EEG and Epilepsy course was organized by Prof. Hans Lüders in Cleveland, OH, in 1979. The course lasted for 8 weeks and included midterm and final examinations for evaluation.^36^ About requirements for successful completion of EEG rotations, responses from US neurology residency program directors ranged from completion of the rotation to passing an oral examination or evaluation and interpretation of 30 EEGs.^30^ However, 64% of neurology residency programs reported not using objective measures to assess EEG milestones.^30^ The basic course on pediatric EEG interpretation sponsored by ICNA includes a self-assessment after completing the learning materials, and a certificate of completion can be downloaded after successfully completing the course.^46^ Even in the programs that use objective measures, there is significant variability in criteria used.^30^ The ACNS In-Service Examination in Clinical Neurophysiology can serve as a certification test to assess knowledge of clinical neurophysiology, including clinical EEG and video-EEG monitoring for clinicians, such as neurology residents.^47^

Most studies focused on evaluating the short-term effectiveness of EEG courses or lectures. Only 3 studies have conducted delayed evaluations. A first study assessed the long-term effectiveness of a face-to-face EEG training program supplemented by e-learning sessions, at 90-day posttraining.^21^ A second study compared pretest and posttest scores in an electronic learning curriculum over a 6-month interval.^23^ A third study evaluated EEG knowledge improvement and retention 12 months after a pretraining examination, following a combination of didactic sessions and on-site teaching.^28^ Despite high variability in baseline knowledge, significant improvements were observed postintervention. The format of precurriculum and postcurriculum tests varied, from single-choice to multiple-choice questions. Some postcurriculum tests used the same format as the precurriculum tests.^3,29^

Regarding the relationship between EEG interpretation competency and PGY in residency, Ding et al.^15^ found that senior adult neurology residents performed significantly better on the ACNS terminology test compared with their junior counterparts after receiving training. Similarly, Nascimento et al.^8^ reported that PGY-4 residents demonstrated superior EEG perception skills compared with PGY-1 to PGY-3 residents, with PGY-3 to PGY-4 residents outperforming PGY-1 to PGY-2 residents. However, Pan et al.^18^ found that the ability to recognize seizures in the EEGs of critically ill patients was not solely dependent on the time spent in epilepsy rotations or the stage of residency training. They suggested that EEG interpretation skills may require an alternate approach and continuous training.

### Residents' Viewpoints About EEG Education

In the most surveys, residents who participated in EEG programs expressed a preference for these programs to become a permanent fixture of their EEG rotations.^32^ According to an online survey of Brazilian neurology residents, 96% of residents reported that learning how to read EEGs during residency was very or extremely important.^31^ However, 43%–45% adult neurology residents were unable to read EEGs even with supervision, and 70% lacked confidence in writing an EEG report independently.^21,31^

From the residents' standpoint, they preferred a combination of didactic lectures and supervised EEG readings as the most efficient way to learn.^26^ They believed that standard EEG lectures were the most effective EEG teaching method, followed by interpreting EEGs under the supervision of attendings.^8,13^ Residents believed that the best measures for ensuring competency were the number of EEGs read and the hours spent in training.^13^ Most reported that reading more than 50 EEGs was necessary for competency.^13^

### Main Barriers in EEG Education

The most commonly reported barriers to EEG learning from residents' view included insufficient exposure, lack of responsibility for reading EEGs during rotations, ineffective didactics, suboptimal supervision from residents or fellows, and inability linking EEG learning to direct patient care.^8,13^ Nascimento et al.^6^ pointed out that from program directors' standpoints, a major barrier was the absence of objective and well-defined EEG competencies expected of graduating residents, including a recognized list of essential EEG findings. Other barriers reported by program directors included excessive inpatient workloads, lack of resident interest, and insufficient time for instruction.^8,30^

The most significant educational barriers, insufficient EEGs exposure, appeared to be related to the short duration of EEG training.^8^ The most common challenges neurology residents experienced in EEG interpretation were recognizing sedative effects, such as drug-induced spindles and identifying focal epileptogenic potentials and slowing.^19^

### EEG Training Provided by Academic Organizations

In recent years, academic organizations such as the ILAE, ACNS, AES, ICNA, CSCN, and CAAE have developed a large number of online and in-person resources for EEG training.^38,46-51^ These programs cater to participants at various levels and cover a wide range of topics, including basic EEG technology, advanced intracranial recordings, pediatric EEG, SEEG workshops, amplitude-integrated EEG, and signal analysis.

The ILAE offers diverse and comprehensive EEG programs and specialized courses (additional data are listed in eTable 1). The ILAE's Virtual Epilepsy Academy (VIREPA) offers basic, advanced, and pediatric EEG courses.^49^ The ILAE Academy provides competency-based curriculum in epileptology, which is divided into 3 levels of professional expertise: level 1—entry, level 2—proficiency, and level 3—advanced proficiency. EEG interpretation is integrated into all levels of the curriculum.^1,39^ The ILAE School on Advanced EEG and Epilepsy offers a hands-on and interactive curriculum for participants with at least 6 months of clinical EEG experience. Topics include signal analysis, seizure semiology, and clinical significance of EEG findings.^49^ The EEG in the first year of life course is a residential program emphasizing interactive and hands-on experience. It focuses on neonates and infants EEG patterns, amplitude-integrated EEG in NICUs, and EEG in genetic and metabolism disorders. The Video-EEG Course on Pediatric Epilepsy includes case presentations and interactive sessions tailored to specialists in pediatric neurology, neuropsychiatry, clinical neurophysiology, and neuropsychology.^49^ The SEEG workshop is a basic, case-based, hands-on course designed for beginners in neurology or neurosurgery, focusing on SEEG technique and interpretation.^49^

Several other academic programs complement EEG training. The Epileptic Disorders Internship Program offers “Roadmap to EEGs,” a series of online educational tools designed to develop competencies in EEG interpretation.^31,40^ The ACNS CNP Bootcamp is a 7-week online program tailored for new fellows in their first weeks of training and residents.^47^ ICNApedia provides an online pediatric EEG course specifically designed for residents.^46^ The AES EEG Learning Curriculum targets junior neurology residents, offering foundational EEG training.^51^ The CAAE Nationwide EEG Training Program combines a 6-month online module (reviewing 30 EEGs biweekly) with face-to-face lectures or video-based modules.^50^ This program is open to participants with at least 1 year of full-time or 2 years of part-time EEG experience.^50^ These programs ensure high-quality training tailored to various expertise levels and subspecialties.

Regarding examinations and certification, most ILAE courses do not require a final examination. However, the ASEPA EEG Part 1 Examination (organized by ILAE-Asia and Oceania) serves as a certification test.^49^ Many VIREPA courses include a clinical case presentation as a final task.^49^ The CSCN conducts annual EEG examinations for eligible physician applicants, primarily tailored for neurology specialists.^48^ The CAAE offers voluntary elementary, intermediate, and advanced-level EEG interpretation examinations.^50^

### Meta-analysis Results

#### Effect of EEG Education on Postcurriculum Test Scores

**Figure 2:**
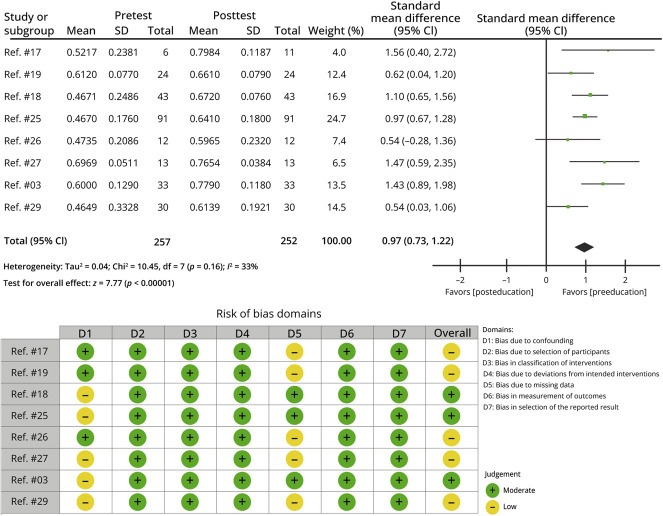
Meta-analysis of the Effect of an Educational Intervention, Using Inverse Variance and a Random-Effects Model, With a Forest Plot Comparing Posteducation and Pre-education Measures, and a Risk of Bias Assessment

An initial meta-analysis of 14 studies revealed a significant improvement in postcurriculum test scores compared with precurriculum test scores, with a pooled SMD of 1.76 (95% CI 1.14–2.39, *p* < 0.00001). However, there was substantial heterogeneity among the studies (τ^2^ = 1.25, χ^2^ = 216.99, *p* < 0.00001, *I*^2^ = 94%). A subgroup analysis of 8 studies focusing on neurology residents showed a pooled SMD of 0.97 (95% CI 0.73–1.22, *p* < 0.00001), with no significant heterogeneity (τ^2^ = 0.04, χ^2^ = 10.45, *p* = 0.16, *I*^2^ = 33%) (Figure 2).

#### Quality of Included Studies

Most studies were ranked as fair to good. Although 2 studies initially were rated as poor according to the NIH Before-After criteria, they were reassessed using the NIH criteria for Observational Studies and received ratings of good and fair, respectively. A risk of bias assessment summarizing the methodologic quality of the included studies within the subgroup is presented in Figure 2.

#### Assessment of Reporting Biases

We did not assess funnel plot asymmetry to detect publication bias because less than 10 studies were included in the subgroup meta-analysis.

### Sensitivity Analysis

A comparative analysis of precurriculum and postcurriculum test scores was conducted by sequentially removing each study and recalculating the significance of the results. The sensitivity analysis showed that the significant heterogeneity observed in the initial analysis could not be fully eliminated. However, it also demonstrated that the overall effect size remained relatively stable when individual studies were excluded from the subgroup of neurology residents, indicating the robustness of the subgroup analysis results.

## Discussion

This systematic review evaluates the effectiveness and current state of EEG education for residents. The results highlight the efficacy of structured EEG training in improving residents' competency, with pooled SMD indicating substantial gains in postcurriculum test scores. Academic organizations play an important role in standardizing and implementing competency-based EEG education. Typically, EEG training is integrated into epilepsy or critical care rotations during residency. Pretests and posttests are vital for evaluating the efficacy of educational programs, while standardized objective assessments ensure accurate measure of residents' competency in EEG interpretation. In addition, greater emphasis should be placed on promoting long-term retention of knowledge following training. Robust EEG education during residency is crucial for preparing future attending physicians with the expertise needed for competent and effective clinical practice.

Currently, most EEGs are interpreted by neurologists without fellowship training in EEG, and there is a lack of consistency in the teaching and evaluation of residents.^24,30^ According to the ACGME milestones for neurology residency, residents are expected to interpret common EEG abnormalities and patterns, recognize normal EEG features and variants, identify common abnormalities and patterns of status epilepticus, and create a report.^3^ Both adult and child neurology residents should achieve full competency in EEG interpretation by the end of their residency, even in the absence of specialized fellowship training.^8^

Some studies believe that traditional didactic lectures were invaluable for resident EEG education,^26,28^ while Schaefer et al.^52^ argued that a purely lecture-based didactic method was particularly ill-suited for teaching EEG interpretation. However, in one Brazilian survey, most residents considered standard EEG lectures the most effective teaching method, followed by supervised EEG interpretation.^13^ Although 96% of residents reported that learning to read EEGs during residency was very or extremely important,^13^ program directors often attributed educational barriers to a perceived lack of residents' interest.^8,30^ This highlights a significant discrepancy in viewpoints between residents and program directors regarding the best way to improve EEG interpretation skills. Traditional training approaches and time-limited rotations may be insufficient for developing proficiency in EEG interpretation. A more structured learning trajectory, postcurriculum feedback, consistency in teaching and evaluation, and regular communication between residents and program directors are essential to ensure long-term effectiveness during residency.

Online courses have become popular since the pandemic. Since on-site places are limited, live-streaming lectures can benefit more interested trainees who cannot participate in person. However, we should know that the efficacy and sustainability of online lectures are limited, particularly those who require hands-on sections.^22^ The effectiveness of EEG training is influenced by the number, qualifications, and dedication of trainers. Certified specialists and qualified EEG technologists are ideal educators for EEG programs.

Collaborative efforts and international partnerships are vital for enhancing EEG education. Resource sharing, such as e-learning modules and online simulators, offers a feasible way to support residency EEG training in resource-limited regions. Competency-based education frameworks ensure that residents acquire essential EEG interpretation skills. Curricula for residents should be tailored to the specific clinical applications of each specialty, with clear outlines of “must-know” EEG knowledge based on the needs of different fields. Some online educational tools such as Roadmap to EEGs can be used as supplement institution-based training methods for adult and child neurology residents.^40^ Implementing conceptual framework such as the IFCN criteria and ACNS critical care EEG terminology offers an efficient self-guided way to learn the basic components of EEG knowledge and markedly improve specificity and agreement among trainees.^15^ Increasing opportunities for hands-on training and in-person experience is crucial for building proficiency in EEG interpretation. Certification programs and continuing education play a critical role in maintaining high standards in this field. Developing standardized EEG education curricula that can be adapted globally is vital to ensure uniform training quality. Enhancing learning motivation, expanding training opportunities, increasing EEGs exposure, and ensuring delayed retention of knowledge are key factors in advancing EEG education for residents. There remains a strong need for more standardized curricula, greater hands-on experience, and comprehensive evaluations to ensure optimal learning outcomes and clinical competence among residents.

EEG education has undergone unprecedented changes, yet there have been few reviews about curriculum characteristics and teaching methods of EEG training for nonepileptologists. Taran et al.^11^ reviewed critical care EEG training for nonneurologists. Kander et al.^41^ focused on EEG training curricula for nonspecialist clinicians. Kromm et al.^42^ examined programs for nonelectroencephalographers in performing and screening adult EEGs. The participants in these reviews were diverse, including fellows, physicians, residents, physician assistants, nurses, technologists, and medical students. Unlike previous reviews, our study included a larger pool of research and specifically focused on EEG programs for residents. The literature we analyzed spanned the past 5 years and reflected the most up-to-date status of EEG education for residents. In addition, we quantitatively compared precurriculum and postcurriculum test scores to provide an objective evaluation of the quality and effectiveness of EEG training programs.

There are some limitations in this review. The sample size of the included residents was relatively small. Although we aimed to reflect the current global status of EEG training for residents, most of the included studies originated from medical advanced countries. In addition, the EEG training programs varied significantly from the identification of IEDs and the use of ACNS Critical Care EEG Terminology to comprehensive EEG training. The content of the training is often depended on the trainers and institutional characteristics. Key aspects, such as the minimum curriculum duration, the required number of EEGs to be interpreted under trainer supervision, and use of objective standardized measures to evaluate competency, remain unclear. The tests in the studies also varied greatly, with some not specifically designed for program evaluation. Moreover, standardized postcurriculum assessments are lacking, and there is limited evaluation of the long-term effectiveness of EEG education for residents. Another limitation is the diversity of residents' specialties included in the studies, which contributed to substantial heterogeneity in the initial meta-analysis. However, this heterogeneity was eliminated when a subgroup analysis focusing on neurology residents was conducted.

Our findings suggest that traditional face-to-face learning, real-time online teaching, and virtual learning are all effective in improving residents' diagnostic accuracy in EEG interpretation. Future research should focus on evaluating the long-term retention of EEG knowledge following training. The development of specialized assessments tailored for EEG program evaluation is essential. Moreover, randomized controlled trials could be conducted to compare different training methodologies to identify the most effective approach for EEG education. To further advance this field, program directors and international academic organizations must collaborate to standardize structured and accessible curricula, and establish consistent evaluation frameworks. These efforts are critical for equipping residents with the skills necessary for proficient EEG interpretation.

This systematic review and meta-analysis highlight the effectiveness of EEG education programs in improving residents' competency. EEG courses combining in-person and e-learning components with sufficient duration should be prioritized to strengthen residents' EEG interpretation skills. Future research should focus on evaluating the long-term retention of EEG knowledge following training, particularly regarding specialties.

## Glossary

ACGME: Accreditation Council for Graduate Medical Education
ACNS: American Clinical Neurophysiology Society
AES: American Epilepsy Society
CAAE: China Association Against Epilepsy
CSCN: Canadian Society of Clinical Neurophysiologists
ICNA: International Child Neurology Association
IED: interictal epileptiform discharge
IFCN: International Federation of Clinical Neurophysiology
ILAE: International League Against Epilepsy
PGY: postgraduate year
SMD: standardized mean difference
